# Reduction in Hypercalcemia Following Readjustment of Target Serum 25-Hydroxy Vitamin D Concentration during Cholecalciferol Therapy in Vitamin D-Deficient Critically Ill Patients

**DOI:** 10.3390/nu14081650

**Published:** 2022-04-15

**Authors:** Roland N. Dickerson, Stephen C. Turner, Whitney L. Holmes, Edward T. Van Matre, Joseph M. Swanson, Saskya Byerly, Dina M. Filiberto, Peter E. Fischer

**Affiliations:** 1 Department of Clinical Pharmacy and Translational Science, University of Tennessee College of Pharmacy, Memphis, TN 38163, USA; edward.vanmatre@uthsc.edu (E.T.V.M.); jswanson@uthsc.edu (J.M.S.); 2 Department of Pharmacy, Mobile Infirmary, Mobile, AL 36607, USA; stephenturner@outlook.com; 3 Department of Pharmacy, Regional One Health, Memphis, TN 38103, USA; wholmes@regionalonehealth.org; 4 Department of Surgery, University of Tennessee Health Science Center, Memphis, TN 38163, USA; sbyerly@uthsc.edu (S.B.); dfiliber@uthsc.edu (D.M.F.); pfischer@uthsc.edu (P.E.F.)

**Keywords:** vitamin D, cholecalciferol, trauma, nutrition, enteral nutrition, vitamins, calcium, critical illness

## Abstract

The intent of this study was an evaluation of our effort to reduce the incidence of hypercalcemia in critically ill vitamin D-deficient patients with multiple traumatic injuries given cholecalciferol. Vitamin D deficiency was defined as a serum 25-hydroxy vitamin D concentration (25-OH vit D) of <20 ng/mL. Adult patients (>17 years of age) were given 10,000 IU of cholecalciferol daily with an intended target 25-OH vit D of >19.9 ng/mL. These patients were compared to a historical control group that underwent therapy with a higher target of >29.9 ng/mL. Patients received cholecalciferol via the feeding tube along with enteral nutrition (EN) until the target 25-OH vit D was achieved, EN discontinued, the nutrition support service signed off the patient, or the patient was discharged from the TICU. Patients were included if two consecutive weekly 25-OH vit D were measured. One hundred and three critically ill trauma patients were retrospectively studied. Fifty were given cholecalciferol therapy with the new lower target 25-OH vit D, and 53 were from a historical cohort aiming for the higher target. Hypercalcemia (serum ionized calcium concentration > 1.32 mmol/L) was reduced from 40% (21 out of 53 patients) to 4% (2 out of 50 patients; *p* < 0.001). None of the hypercalcemic patients were symptomatic. Readjustment of target 25-OH vit D concentration resulted in a ten-fold decrease in the rate of hypercalcemia and improved the safety of cholecalciferol therapy for critically ill patients with traumatic injuries.

## 1. Introduction

Vitamin D deficiency has been associated with increased morbidity, increased length of stay, and mortality among critically ill patients [1,2,3,4,5,6,7]. Our previous data indicated that three-fourths of critically ill trauma patients are vitamin D deficient [8]. A pivotal paper that reanalyzes data derived from the VitDAL-ICU trial [9] may provide further insight as to whom will benefit from cholecalciferol therapy [10]. Of the original 475 study participants from the VitDAL-ICU trial, the investigators omitted 65 patients who either died or were discharged from the ICU within the first week of ICU admission. In this subpopulation, the data indicate that cholecalciferol therapy is associated with improved 28-day mortality [10]. These data would infer that those with prolonged intensive care unit (ICU) stays and suffering from persistent inflammation, immunosuppression, and catabolism syndrome might benefit from vitamin D therapy. Our critically ill trauma patients often require prolonged enteral nutrition (EN), undergo multiple surgical procedures, and many exhibit evidence of vitamin D deficiency [8]. Additionally, this population is at high risk for infections, exhibit prolonged elevations in serum C-reactive protein concentrations, persistently catabolic, and have an average ICU length of stay from three to four weeks [11,12,13]. Thus, this population may be a group that could potentially benefit from vitamin D therapy [8,14,15,16].

Based on this argument, until the data are clearer, we have opted to treat vitamin D deficiency in our critically ill patients with traumatic injuries who receive EN if it can be done safely. Our previous investigation of vitamin D therapy for critically ill vitamin D-deficient patients was designed with the intent of achieving a serum 25-OH vit D within the normal range (30 to 80 ng/mL) [15]. However, despite a lack of hypervitaminosis D (25-OH vit D > 80 ng/mL), 40% of the patients experienced hypercalcemia (serum ionized calcium concentration (iCa) > 1.32 mmol/L) during cholecalciferol therapy [15]. The purpose of this study was to ascertain if lowering the target 25-OH vit D to ≥20 ng/mL (to ameliorate vitamin D deficiency versus achievement of serum concentrations observed in healthy subjects) would also reduce the incidence of hypercalcemia during cholecalciferol therapy.

## 2. Materials and Methods

Adult patients with vitamin D deficiency (25-OH vit D < 20 ng/mL) [17,18], admitted to the trauma ICU, and given 10,000 IU of liquid cholecalciferol daily were evaluated. The initial 25-OH vit D was obtained at least 2 days following ICU admission to avoid serum concentration aberrations following fluid resuscitation [19] and weekly thereafter. Serum 25-OH vit D was determined via a chemiluminescent microparticle immunoassay [20,21]. Cholecalciferol therapy was discontinued when the target 25-OH vit D was achieved or when the patient was discharged from the trauma ICU, enteral nutrition therapy was discontinued, the nutrition support service signed off the case, or if the patient experienced hypercalcemia.

Patients were intragastrically fed via a feeding tube when hemodynamically stable following fluid resuscitation. Commercially available enteral formulas were given to all patients. These formulas contained 400–533 IU of vitamin D_3_ and 667–800 mg of elemental calcium per 1000 kcals. Assigned calorie and protein goals were 30 to 32 kcal/kg/day and 2 to 2.5 g/kg/day, respectively [11]. Those with obesity (body mass index ≥ 30 kg/m^2^) were given ≤25 kcal/kg/d and 2 to 2.5 g/kg/d based on ideal body weight [13,22,23]. For patients who required an intravenous propofol infusion, the EN regimen was altered to avoid caloric overfeeding from the combined lipid content of propofol with enteral feeding as previously described [24]. Pre-admission or pre-resuscitation body weight was used for nutritional computations when possible. Patients were given an immuno-modulating EN regimen if the patient’s estimated injury severity score (ISS) [25] was greater than 20 and if the patient did not have an infection [26]. The enteral feeding rate was advanced over two to four days as tolerated to achieve the goal regimen. Prokinetic agents (metoclopramide or combined metoclopramide and erythromycin therapy) were used if the patient experienced gastric feeding intolerance [27]. Liquid protein supplements were given with the continuous EN to meet increased protein goals for this population [11,13]. Glycemic control was achieved by administration of different modes of insulin therapy to achieve target blood glucose concentrations of 70 to 150 mg/dL if necessary [28,29,30]. 

Patients excluded from the study were those who received pamidronate, etidronate, long-term administration of corticosteroids, therapeutic doses of various congeners of vitamin D, or those who were receiving an ad libitum oral diet in lieu of continuous EN. Patients who received parenteral nutrition for ≥10 days were also excluded due to their inability to receive enterally administered medications for an extended time. Those with kidney failure requiring dialysis or who had a medical history of bone disease, chronic granulomatous diseases, or who were pregnant were also excluded.

Patients were identified from the Nutrition Support Service records, and the patients’ hospital electronic charts were retrospectively reviewed. These data were then compared to a historical cohort of critically ill patients with traumatic injuries who also received cholecalciferol therapy following evidence of vitamin D deficiency [15]. Patients from the historical cohort underwent the same inclusion and exclusion criteria as the current protocol apart from the target 25-OH vit D. Target 25-OH vit D for the previously published historical cohort [15] was set at ≥30 ng/mL which is considered the lower limit of the normal range for healthy subjects [17]. Serum ionized calcium concentration was used to assess the presence of hypercalcemia since the relationship between total calcium concentrations and predictive ionized calcium concentrations based solely on serum albumin concentrations are altered in critically ill patients [31,32]. This retrospective study was approved by the university Institutional Review Board and the hospital Office of Medical Research. The requirement for written informed consent was waived.

SigmaPlot for Windows, version 14.5 (Systat Software, Point Richmond, VA, USA) was used for statistical analysis. A probability value of <0.05 was defined as statistically significant. The Student’s t-test or Mann–Whitney rank sum test were used to compare the two groups depending on normality of the data. Data are represented as median (25%, 75% quartile ranges). Nominal data were analyzed by chi square or the Fisher’s exact test. Two-way ANOVA with post hoc pair-wise comparisons by the Student–Newman–Keuls method was used for comparing serial measurements between groups over time.

## 3. Results

A total of 103 critically ill ventilator-dependent patients admitted to the TICU were evaluated. Most patients were male (*n* = 73, 71%), white (*n* = 53, 51%), admitted due to a motor vehicle collision (*n* = 77, 75%), and survived (*n* = 94, 91%). Most patients had multiple traumatic injuries, and over half experienced severe traumatic brain injury and were adequate in body weight (BMI > 18.5 kg/m^2^), overweight (BMI > 25 kg/m^2^), or obese (BMI > 30 kg/m^2^). Other patient characteristics are given in Table 1. Patients demonstrated a systemic inflammatory response with a markedly increased serum C-reactive protein concentration (CRP), elevated white blood cell count (WBC), and decreased serum prealbumin concentration (Table 2). Patients had normal renal function as evidenced by serum creatinine and urea nitrogen concentrations (Table 2).

Fifty patients received cholecalciferol therapy with a targeted 25-OH vit D of ≥20 ng/mL, and 53 patients were from a historical consort of patients [15] whereby a 25-OH vit D of ≥30 ng/mL was targeted. There was no difference in patient characteristics between groups with respect to age, sex, race, weight, BMI, admission diagnosis, presence of TBI, furosemide or hydrochlorothiazide exposure (Table 1). Enteral nutrition therapy was started on hospital day 2 (1, 3) for both groups. Baseline serum total calcium, phosphorus, and magnesium concentrations were not significantly different between target groups. Other baseline laboratories are given in Table 2.

**Figure 1:**
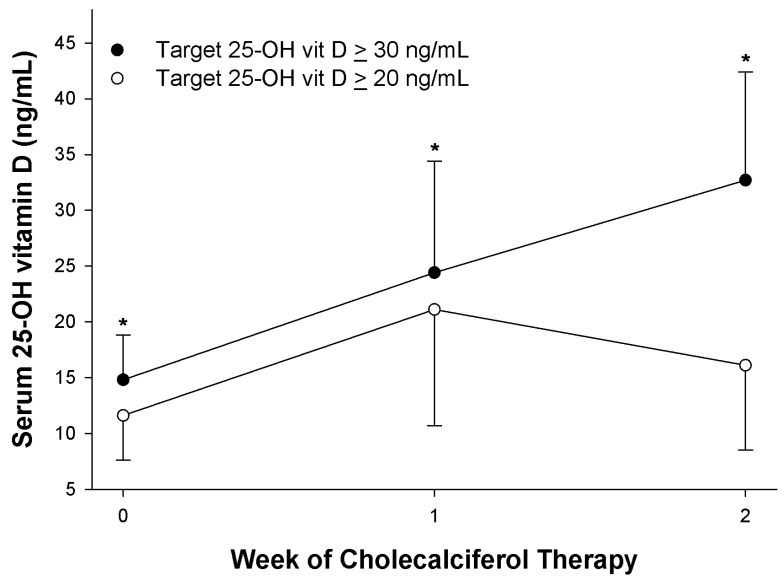
Serum 25-hydroxy vitamin D response to cholecalciferol therapy between the lower target (25-OH vit D ≥ 20 ng/mL) and higher target (25-OH vit D ≥ 30 ng/mL) groups, respectively. Week 2 concentrations were from 21 and 29 patients from the lower and higher 25-OH vit D target groups, respectively. Data given as mean + S.D. * *p* < 0.05 between target groups by week of therapy.

Cholecalciferol dosing characteristics are given in Table 3. Mean serum 25-OH vit D was obtained, on average, on TICU day 5, for both groups. Cholecalciferol therapy was initiated 1–2 days after receipt of a 25-OH vit D < 20 ng/mL and continued for 8 (7, 14) days for the lower target group compared to 13 (10, 17) days for the higher target group (*p* = 0.003). Nearly three fourths of the lower 25-OH vit D target group experienced 25-OH vit D ≥ 20 ng/mL in contrast to over 90% for the higher target group (*p* = 0.005). Only 20% of patients in the lower target group exceeded 29.9 ng/mL compared to about half of the higher target group (*p* = 0.004). Figure 1 illustrates 25-OH vit D response to cholecalciferol therapy for both groups. The higher target group had a progressive increase in 25-OH vit D from baseline to weeks 1 and 2 with a significantly greater 25-OH vit D concentration than the lower target group at each time interval (*p* < 0.05; Figure 1). However, these data are also supported by an earlier discontinuation of cholecalciferol therapy for the lower target group. As a result, week 2 only represents 40% of patients from the lower target group who required continued monitoring and cholecalciferol therapy (Figure 1), as therapy and monitoring were discontinued in the others.

**Figure 2:**
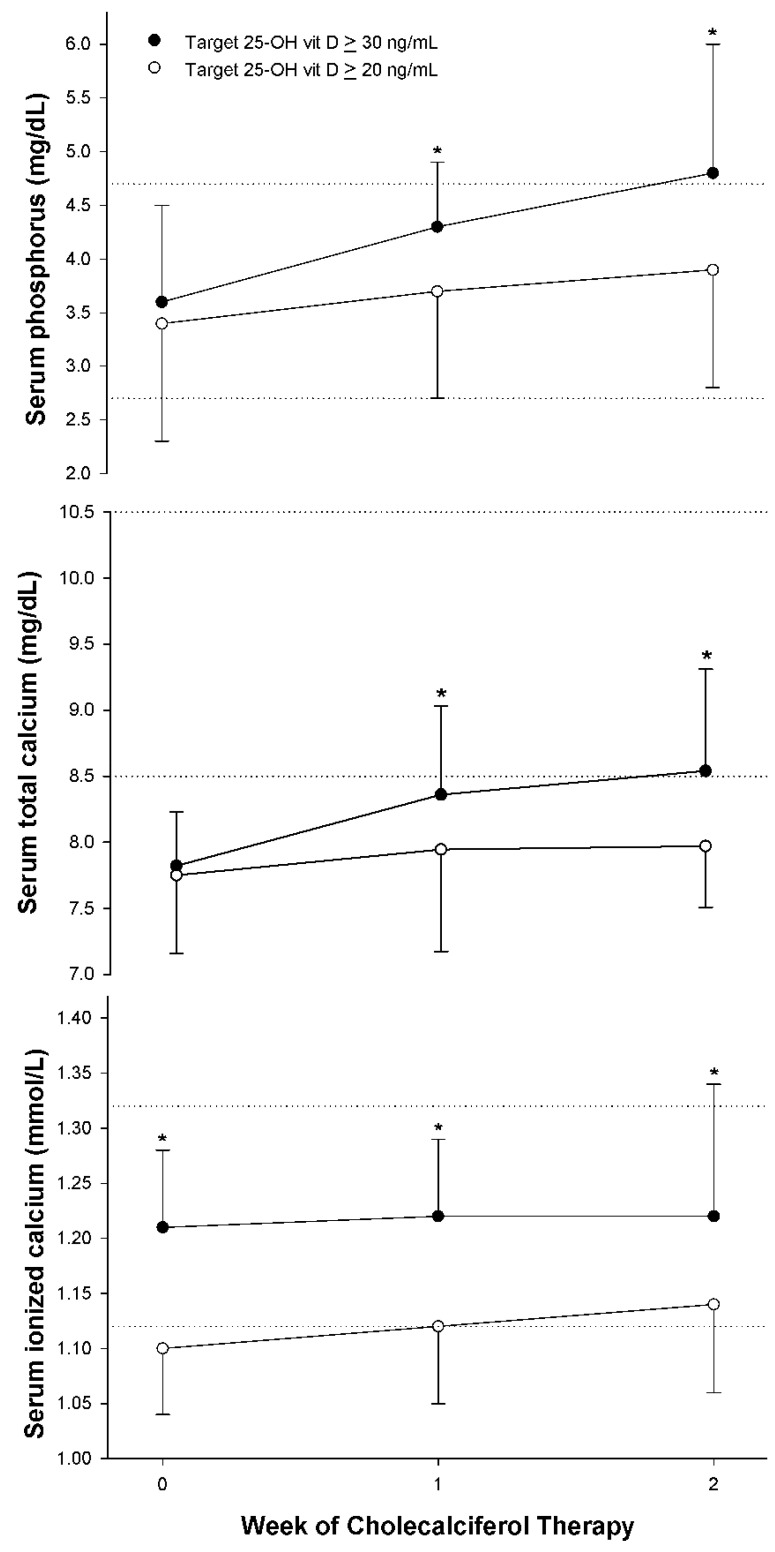
Serial serum ionized calcium, total calcium, and phosphorus concentrations during cholecalciferol therapy between the higher target (25-OH vit D ≥ 30 ng/mL) and lower target (25-OH vit D ≥ 20 ng/mL) groups, respectively. The dotted lines represent normal serum concentration range. Data given as mean + S.D.* *p* < 0.05 between target groups by week of therapy.

Reducing the target 20-OH vit D from ≥30 to ≥20 ng/mL led to a ten-fold reduction in hypercalcemia from 40% to 4% (Table 4). All hypercalcemic patients were asymptomatic. This difference in hypercalcemic rates between target groups could not be explained by differences in patient demographics (Table 1 and Table 2), as both patient populations were derived from the same TICU over two different time periods. As anticipated, the time to achieve the maximum iCa occurred later for the higher target group (16 (13, 21) days vs. 13 (8, 16) days, *p* = 0.003) as those in the higher target group received cholecalciferol for a longer duration. However, these findings were potentially confounded by an unexplainable lower baseline 25-OH vit D for the lower target group and higher baseline iCa for the higher target group (Table 4, Figure 1 and Figure 2). Although mean iCa rose weekly over time with cholecalciferol therapy for the lower target group, iCa concentration remained consistently lower than that in the higher target group for each weekly observation (Figure 2). Serum total calcium concentrations tended to parallel ionized calcium concentrations (Figure 2). Serum phosphorus concentrations also rose over time for both groups (Figure 2). Despite similar baseline serum concentrations, the higher target dosing group had significantly higher serum phosphorus concentrations by weeks 1 and 2 of cholecalciferol therapy (Figure 2). Forty-eight percent of the lower target group developed hyperphosphatemia (serum phosphorus concentration ≥5 mg/dL) compared to 64% of the higher target group (*p* = 0.115).

## 4. Discussion

The presence of vitamin D deficiency in critically ill patients has historically been associated with increased morbidity and increased mortality [1,2,3,4,5,6,7]. We have previously shown that three-fourths of critically ill trauma patients with severe traumatic injuries who have a prolonged length of stay in the TICU and require prolonged enteral tube feeding are vitamin D deficient [8]. Conflicting studies pose the query whether vitamin D supplementation can improve morbidity and mortality for critically ill patients [9,10,31]. However, it is difficult to come to a clear conclusion, as recent studies are confounded by numerous factors, including heterogenous patient populations, inadequate number of patients, varying vitamin D dosages, dosing intervals, parenteral versus enteral routes of administration, and different congeners of vitamin D. In a reanalysis of the VitDAL-ICU trial [9], critically ill patients who did not expire within the first seven days of ICU admission required an ICU duration of stay of at least a week, and given a large single dosage of cholecalciferol (540,000 IU) experienced a reduction in mortality when compared to those who received placebo [10]. This study supports our premise that, until further evidence is available to indicate otherwise, those with prolonged ICU stays and suffering from persistent inflammation, immunosuppression, and catabolism syndrome, such as critically ill patients with severe traumatic injuries [11,12,13], might benefit from cholecalciferol therapy as long as it can be done safely. 

The optimum dosage of cholecalciferol for critically ill patients with multiple traumatic injuries is unknown. We were concerned regarding the safety of a large single dose (540,000 IU) of cholecalciferol in addition to the practicalities of its administration in hospitalized ICU patients via a feeding tube. The European Endocrine Society guidelines suggest that doses up to 10,000 IU daily for several months are well-tolerated without adverse effects (e.g., hypercalcemia) [17]. We anticipated that patients would be given 10,000 IU of commercially available liquid cholecalciferol (400 IU/mL for a total of 25 mL) daily per the feeding tube until the target 25-OH vit D was achieved. We anticipated that patients would require an overall duration of vitamin D therapy lasting one to three weeks. Our previous study with cholecalciferol with a target 25-OH vit D of ≥30 ng/mL [32] served as the historical control group for this study. Serum 25-OH vit D rose steadily from baseline up to two weeks of therapy (Figure 1) with 92% of patients achieving a 25-OH vit D ≥ 20 ng/mL, and half of the patients achieving target 25-OH vit D > 30 ng/mL by the second week of therapy (Table 3). Surprisingly, 40% of patients exhibited asymptomatic hypercalcemia (iCa > 1.32 mmol/L) [32], which prompted a re-evaluation of our dosing strategy to a reduced target 25-OH vit D goal of ≥20 ng/mL. This new target was chosen as an acceptable goal, as worsened clinical outcomes have only been associated with 25-OH vit D < 20 ng/mL [1,2,3,4,5,6,7]. In addition, we anticipated that this lower target goal would result in a lower rate of hypercalcemia. Cholecalciferol dosing with the new lower target goal exhibited a similar rate of rise in 25-OH vit D after one week of treatment as the higher target group (Figure 1). As anticipated, the lower target group received a shorter duration of cholecalciferol therapy than the higher target group (Table 3). After two weeks, 70% of the patients from the lower target treatment arm achieved the target 25-OH vit D > 20 ng/mL, and 20% were >30 ng/mL (Table 3). Although 25-OH vit D appeared to decline by the second week (Figure 1), this was reflective of a lower number of observations (21 out of 50 patients) due to intentional earlier discontinuation of cholecalciferol therapy secondary to achievement of the lower 25-OH vit D goal. Thus, those remaining patients in the second observation week predominantly comprised those who had not yet achieved the goal of 25-OH vit D. In this lower 25-OH vit D treatment arm, only 4% of patients developed hypercalcemia (*p* = 0.001 when compared to the higher target group; Table 4). 

The etiologies for why a substantial proportion of patients developed hypercalcemia in the historical higher-target 25-OH vit D treatment arm [15] is not entirely clear and likely multifactorial. Despite hypercalcemia, hypervitaminosis D (25-OH vit D > 79.9 ng/mL) [17] was not evident in any of the patients. In one study of 475 serum samples with an elevated 25-OH vit D within 64–455 ng/mL, only 10% of the samples exhibited hypercalcemia [33]. Others have argued that hypercalcemia may not occur from hypervitaminosis D until 25-OH vit D is consistently above 150 ng/mL [34,35]. These rates of hypercalcemia are significant given the low incidence of hypercalcemia for the large single cholecalciferol dosage trials (VIOLET [36] and VitDAL-ICU [9]). The VIOLET trial [36] was 2.9% at day 14 (vs. 1.9% for placebo) and 1.4% (vs. 0% for placebo) at day 28 for the VitDAL-ICU trial [9]. Unlike the VIOLET and VitDAL-ICU trials, patients from our institution suffered from multiple fractures requiring surgical orthopedic interventions, and it could be hypothesized that calcium homeostasis aberrations occurred due to extensive bone injuries and repair. Immobilization may also be implicated as a contributing to factor to hypercalcemia as immobilization has led to bone hyper-resorption and hypercalcemia in chronic critically ill patients with prolonged hospitalization [37]. Some of the patients in both 25-OH vit D target treatment arms in this study were immobilized via skeletal traction to stabilize and realign bone fractures. However, the appearance of hypercalcemia is uncommon while patients are in the TICU prior to transfer to a stepdown unit or the floor. Prior to initiation of vitamin D therapy at our institution, only 6 of 100 consecutive patients (6%) admitted to the TICU with severe traumatic injuries exhibited hypercalcemia [31]. Thus, bone injuries and immobilization for some patients could not explain the marked rate of hypercalcemia observed in our historical control group.

We suspect the greater incidence of hypercalcemia for the higher target group was attributed to achievement of a higher 25-OH vit D, a more prolonged duration of cholecalciferol therapy, and a higher baseline 25-OH vit D and greater baseline iCa than that of the lower 25-OH vit D target group. Our previous investigation indicated that those who developed hypercalcemia had a significantly greater 25-OH vit D response by the second week of therapy than those who did not experience hypercalcemia [15]. From this study, more patients in the higher-target group achieved 25-OH vit D > 20 and 30 ng/mL (Table 3, Figure 1). The lower-target 25-OH vit D group may have experienced greater vitamin D deficiency as indicated by a lower 25-OH vit D prior to initiation of cholecalciferol therapy (Table 4, Figure 1). Development of hypercalcemia as well as significant increases in serum phosphorus concentrations without hypervitaminosis D (Table 4, Figure 2) suggested that total 25-OH vit D may be limited as a marker for adequate repletion. Vitamin D is bound to and carried by vitamin D binding protein (VDBP) and albumin. However, the systemic inflammatory response to critical illness is associated with a decrease in VDBP and serum transport proteins such as albumin [38]. Patients in this study had significant elevations in serum CRP concentrations and depressed serum prealbumin concentrations, indicating a high level of inflammatory stress. As a result, it is theoretically plausible that saturation of binding sites may have occurred, resulting in a greater proportion of bioavailable 25-OH vit D and 1.25 di hydroxy vit D despite “normal” 25-OH vit D. Potential aberrations in protein binding theoretically could have possibly led to exaggerated vitamin D expression, resulting in hypercalcemia and hyperphosphatemia [34,39]. Unfortunately, neither free 25-OH vit D nor VDBP concentrations were available in our hospital laboratory to verify this hypothesis. Finally, it is also possible that the higher 25-OH vit D target group was at greater risk of developing hypercalcemia, as baseline iCa was in the midpoint of the normal range and significantly greater than the lower target group (Figure 2).

This study has limitations. It was a retrospective, single-center study with a limited number of patients. Our definition of vitamin D deficiency was a 25-OH vit D < 20 ng/mL; however, some other international societies provide a lower threshold concentration of ≤12 ng/mL [40]. Lack of determination of VDBP, free 25-OH vit D, free and total 1.25 di hydroxy vit D, vitamin D metabolites, and parathyroid hormone concentration could have provided further mechanistic insight for the etiology of hypercalcemia. Steady-state 25-OH vit D measurements were unlikely due to day-to-day variability in the clinical status of the patients, a short two-week evaluation, as well as a limited amount of dosage omissions. Finally, it is still controversial whether vitamin D therapy improves clinical outcomes for vitamin D-deficient critically ill patients. The ongoing VITDALIZE study is a randomized, placebo-controlled international trial in a projected 2400 critically ill adult patients with vitamin D deficiency that will attempt to answer this most important issue [41].

## 5. Conclusions

Reduction in target 25-OH vit D improved the safety profile for cholecalciferol therapy for critically ill patients with severe traumatic injuries while achieving 25-OH vit D ≥ 20 ng/mL for most patients. Severity of vitamin D deficiency and baseline serum ionized calcium concentrations should also be evaluated when planning cholecalciferol therapy for these patients. The higher 25-OH vit D target of >29.9 ng/mL is not recommended. Further evaluation, especially in consideration of whether vitamin D therapy can improve clinical outcomes, is warranted.

## Figures and Tables

**Table 1:** Patient characteristics.

Variable	Serum 25-OH vit D Target		*p*
	≥20 ng/mL	≥30 ng/mL	
*N*	50	53	-
Age, years	46 (30, 62)	42 (31, 56)	0.404
Sex, male, *n* (%)	37 (74%)	36 (68%)	0.498
Race, *n* (%)			
White	26 (52%)	27 (51%)	
African American	22 (44%)	23 (43%)	0.228
Hispanic/Other	2 (4%)	3 (6%)	
Weight, kg	84 (71, 102)	85 (72, 102)	0.992
BMI, kg/m^2^	26.5 (23.4, 31.8)	26.9 (23.1, 31.7)	0.805
Admission Diagnosis			
MVC, *n* (%)	38 (76%)	39 (75%)	
GSW/KSW, *n* (%)	6 (12%)	6 (11%)	
Assault/Fall, *n* (%)	6 (12%)	4 (8%)	0.228
Other, *n* (%)	0 (0%)	4 (8%)	
TBI, *n* (%)	32 (64%)	31 (58%)	0.566
Alcohol use, *n* (%)	15 (30%)	11 (21%)	0.164
Hospital day EN started	2 (1, 3)	3 (2, 3)	0.085
Initial Tmax, °C	39.0 (38.8, 39.5)	38.1 (37.5, 38.6)	0.001
Sepsis, *n* (%)	23 (46%)	31 (58%)	0.205
Survived, *n* (%)	45 (90%)	49 (92%)	0.660
Furosemide exposure, *n* (%)	22 (44%)	20 (38%)	0.518
HCTZ exposure, *n* (%)	5 (10%)	2 (4%)	0.210

BMI, body mass index; EN, enteral nutrition; GSW, gunshot wound; HCTZ, hydrochlorothiazide; KSW, knife stab wound; MVC, motor vehicle collision; *N/n*, number of patients; TBI, traumatic brain injury; Tmax, maximum temperature; WBC, white blood cells.

**Table 2:** Baseline laboratories.

Variable	Serum 25-OH vit D Target		*p*
	≥20 ng/mL	≥30 ng/mL	
*N*	50	53	
WBC, cells/mm^3^	17.4 (14.8, 21.9)	16.9 (12.2, 20.0)	0.229
C-reactive protein, mg/dL	19.4 (14.4, 24.0)	25.2 (18.9, 29.3)	0.023
Prealbumin, mg/dL	8.0 (6.0, 11.4)	6.8 (4.4, 9.9)	0.115
Urea nitrogen, mg/dL	14 (9, 26)	14 (9, 17)	0.344
Creatinine, mg/dL	0.8 (0.6, 1.0)	0.8 (0.6, 0.9)	0.868
Total calcium, mg/dL	7.8 (7.4. 8.0)	7.8 (7.6, 8.1)	0.392
mmol/L	1.95 (1.86, 2.00)	1.95 (1.90, 2.02)	
Phosphorus, mg/dL	3.3 (2.6, 4.3)	3.4 (2.8, 4.3)	0.325
Magnesium, mg/dL	2.0 (1.8, 2.2)	2.1 (1.9, 2.2)	0.214

25-OH vit D, 25-hydroxy vitamin D; *N*, number of patients; WBC, white blood cell count.

**Table 3:** Cholecalciferol therapy.

Variable	Serum 25-OH vit D Target		*p*
	≥20 ng/mL	≥30 ng/mL	
*N*	50	53	
Initial determination of 25-OH vit D, days	4 (2, 6)	4 (3, 6)	0.205
Hospital day cholecalciferol initiated, days	5 (3, 8)	6 (4, 8)	0.365
Duration of cholecalciferol therapy, days	8 (7, 14)	13 (10, 17)	0.003
Achieved 25-OH vit D ≥ 20 ng/mL, *n* (%)	35 (70%)	49 (92%)	0.005
Achieved 25-OH vit D ≥ 30 ng/mL, *n* (%)	10 (20%)	26 (49%)	0.004

25-OH vit D, 25-hydroxy vitamin D; *N/n*, number of patients.

**Table 4:** Pathogenesis of hypercalcemia.

Variable	Serum 25-OH vit D Target		*p*
	≥20 ng/mL	≥30 ng/mL	
*N*	50	53	
Hypercalcemia (iCa > 1.32 mmol/L), *n*	2 (4%)	21 (40%)	0.001
iCa prior to cholecalciferol, mmol/L	1.10 (1.06, 1.14)	1.19 (1.15, 1.22)	0.001
mg/dL	4.4 (4.3, 4.6)	4.9 (4.7, 5.0)	
Baseline 25-OH vit D, ng/mL	11.0 (8.0, 14.7)	13.0 (13.0, 15.9)	0.001
Maximum iCa, mmol/L	1.15 (1.12, 1.18)	1.30 (1.23, 1.34)	0.001
mg/dL	4.6 (4.5, 4.7)	5.2 (5.0, 5.4)	
Hospital day to achieve maximum iCa, days	13 (8, 16)	16 (13, 21)	0.004
Number of cholecalciferol doses given at maximum iCa	5 (1, 8)	7 (4, 12)	0.012

25-OH vit D, 25-hydroxy vitamin D; iCa, serum ionized calcium concentration, *N*/*n*, number of patients.

## Data Availability

The dataset used and analyzed for the current study is available from the corresponding author upon reasonable request.

## References

[1] Amrein K., Zajic P., Schnedl C., Waltensdorfer A., Fruhwald S., Holl A., Purkart T.U., Wünsch G., Valentin T., Grisold A. (2014). Vitamin D status and its association with season, hospital and sepsis mortality in critical illness. Crit. Care.

[2] Braun A., Chang D., Mahadevappa K., Gibbons F.K., Liu Y., Giovannucci E., Christopher K.B. (2011). Association of low serum 25-hydroxyvitamin D levels and mortality in the critically ill. Crit. Care Med..

[3] Braun A.B., Gibbons F.K., Litonjua A.A., Giovannucci E., Christopher K.B. (2012). Low serum 25-hydroxyvitamin D at critical care initiation is associated with increased mortality. Crit. Care Med..

[4] Higgins D.M., Wischmeyer P.E., Queensland K.M., Sillau S.H., Sufit A.J., Heyland D.K. (2012). Relationship of vitamin D deficiency to clinical outcomes in critically ill patients. JPEN J. Parenter. Enteral. Nutr..

[5] Moromizato T., Litonjua A.A., Braun A.B., Gibbons F.K., Giovannucci E., Christopher K.B. (2014). Association of low serum 25-hydroxyvitamin D levels and sepsis in the critically ill. Crit. Care Med..

[6] Quraishi S.A., Bittner E.A., Blum L., McCarthy C.M., Bhan I., Camargo C.A. (2014). Prospective study of vitamin D status at initiation of care in critically ill surgical patients and risk of 90-day mortality. Crit. Care Med..

[7] Venkatram S., Chilimuri S., Adrish M., Salako A., Patel M., Diaz-Fuentes G. (2011). Vitamin D deficiency is associated with mortality in the medical intensive care unit. Crit. Care..

[8] Dickerson R.N., Van Cleve J.R., Swanson J.M., Maish G.O., Minard G., Croce M.A., Brown R.O. (2016). Vitamin D deficiency in critically ill patients with traumatic injuries. Burn. Trauma.

[9] Amrein K., Schnedl C., Holl A., Riedl R., Christopher K.B., Pachler C., Purkart T.U., Waltensdorfer A., Münch A., Warnkross H. (2014). Effect of high-dose vitamin D3 on hospital length of stay in critically ill patients with vitamin D deficiency: The VITdAL-ICU randomized clinical trial. JAMA..

[10] Martucci G., McNally D., Parekh D., Zajic P., Tuzzolino F., Arcadipane A., Christopher K.B., Dobnig H., Amrein K. (2019). Trying to identify who may benefit most from future vitamin D intervention trials: A post hoc analysis from the VITDAL-ICU study excluding the early deaths. Crit. Care..

[11] Dickerson R.N., Pitts S.L., Maish G.O., Schroeppel T.J., Magnotti L.J., Croce M.A., Minard G., Brown R.O. (2012). A reappraisal of nitrogen requirements for patients with critical illness and trauma. J. Trauma Acute Care Surg..

[12] Dickerson R., Crawford C., Tsiu M., Bujanowski C., Van Matre E., Swanson J., Filiberto D., Minard G. (2021). Augmented renal clearance following traumatic injury in critically ill patients requiring nutrition therapy. Nutrients.

[13] Dickerson R.N., Medling T.L., Smith A.C., Maish G.O., Croce M.A., Minard G., Brown R.O. (2013). Hypocaloric, high-protein nutrition therapy in older vs younger critically ill patients with obesity. JPEN J. Parenter. Enteral. Nutr..

[14] Dickerson R.N., Berry S.C., Ziebarth J.D., Swanson J.M., Maish G.O., Minard G., Brown R.O. (2015). Dose-response effect of ergocalciferol therapy on serum 25-hydroxyvitamin D concentration during critical illness. Nutrition.

[15] Holmes W.L., Maish G.O., Minard G., Croce M.A., Dickerson R.N. (2020). Hypercalcemia without hypervitaminosis D during cholecalciferol supplementation in critically ill patients. Nutr. Clin. Pract..

[16] Dickerson R.N., Holmes W.L., Maish G.O., Croce M.A., Minard G. (2019). Obesity attenuates serum 25-hydroxyvitamin D response to cholecalciferol therapy in critically ill patients. Nutrition.

[17] Holick M., Binkley N.C., Bischoff-Ferrari H., Gordon C.M., Hanley D.A., Heaney R.P., Murad M.H., Weaver C.M. (2011). Evaluation, treatment, and prevention of vitamin D deficiency: An Endocrine Society clinical practice guideline. J. Clin. Endocrinol. Metab..

[18] Matthews L.R., Ahmed Y., Wilson K.L., Griggs D.D., Danner O.K. (2012). Worsening severity of vitamin D deficiency is associated with increased length of stay, surgical intensive care unit cost, and mortality rate in surgical intensive care unit patients. Am. J. Surg..

[19] Krishnan A., Ochola J., Mundy J., Jones M., Kruger P., Duncan E., Venkatesh B. (2010). Acute fluid shifts influence the assessment of serum vitamin D status in critically ill patients. Crit. Care.

[20] Farrell C.J., Martin S., McWhinney B., Straub I., Williams P., Herrmann M. (2012). State-of-the-art vitamin D assays: A comparison of automated immunoassays with liquid chromatography-tandem mass spectrometry methods. Clin. Chem..

[21] Abbott Laboratories (2011). 25-OH Vitamin D Architect System Package Insert.

[22] Dickerson R.N., Boschert K.J., Kudsk K.A., Brown R.O. (2002). Hypocaloric enteral tube feeding in critically ill obese patients. Nutrition.

[23] Choban P., Dickerson R., Malone A., Worthington P., Compher C., American Society for Parenteral and Enteral Nutrition (2013). ASPEN Clinical guidelines: Nutrition support of hospitalized adult patients with obesity. JPEN J. Parenter Enteral. Nutr..

[24] Buckley C.T., Van Matre E.T., Fischer P.E., Minard G., Dickerson R.N. (2021). Improvement in protein delivery for critically ill patients requiring high-dose propofol therapy and enteral nutrition. Nutr. Clin. Pract..

[25] Baker S.P., O’Neill B., Haddon W., Long W.B. (1974). The injury severity score: A method for describing patients with multiple injuries and evaluating emergency care. J. Trauma..

[26] Kudsk K.A., Minard G., Croce M.A., Brown R.O., Lowrey T.S., Pritchard F.E., Dickerson R., Fabian T.C. (1996). A randomized trial of isonitrogenous enteral diets after severe trauma. An immune-enhancing diet reduces septic complications. Ann. Surg..

[27] Dickerson R.N., Mitchell J.N., Morgan L.M., Maish G.O., Croce M.A., Minard G., Brown R.O. (2009). Disparate response to metoclopramide therapy for gastric feeding intolerance in trauma patients with and without traumatic brain injury. JPEN J. Parenter Enteral. Nutr..

[28] Cogle S.V., Smith S.E., Maish G.O., Minard G., Croce M.A., Dickerson R.N. (2017). Sliding scale regular human insulin for identifying critically ill trauma patients who require intensive insulin therapy and for glycemic control in those with mild to moderate hyperglycemia. J. Pharm. Nutr. Sci..

[29] Dickerson R.N., Swiggart C.E., Morgan L.M., Maish G.O., Croce M.A., Minard G., Brown R.O. (2008). Safety and efficacy of a graduated intravenous insulin infusion protocol in critically ill trauma patients receiving specialized nutritional support. Nutrition.

[30] Dickerson R.N., Wilson V.C., Maish G.O., Croce M.A., Minard G., Brown R.O. (2013). Transitional NPH insulin therapy for critically ill patients receiving continuous enteral nutrition and intravenous regular human insulin. JPEN J. Parenter Enteral. Nutr..

[31] Dickerson R.N., Alexander K.H., Minard G., Croce M.A., Brown R.O. (2004). Accuracy of methods to estimate ionized and “corrected” serum calcium concentrations in critically ill multiple trauma patients receiving specialized nutrition support. JPEN J Parenter Enteral. Nutr..

[32] Dickerson R.N., Henry N.Y., Miller P.L., Minard G., Brown R.O. (2007). Low serum total calcium concentration as a marker of low serum ionized calcium concentration in critically ill patients receiving specialized nutrition support. Nutr. Clin. Pract..

[33] Ginde A.A., Brower R.G., Caterino J.M., Finck L., Banner-Goodspeed V.M., Grissom C.K., Hayden D., Hough C.L., Hyzy R.C., Khan A. (2019). Early high-dose vitamin D3 for critically ill, vitamin D-deficient patients. N. Engl. J. Med..

[34] Perez-Barrios C., Hernandez-Alvarez E., Blanco-Navarro I., Perez-Sacristan B., Granado-Lorencio F. (2016). Prevalence of hypercalcemia related to hypervitaminosis D in clinical practice. Clin. Nutr..

[35] Jones G. (2008). Pharmacokinetics of vitamin D toxicity. Am. J. Clin. Nutr..

[36] Galior K., Grebe S., Singh R. (2018). Development of vitamin D toxicity from overcorrection of vitamin D deficiency: A review of case reports. Nutrients.

[37] Nierman D.M., Mechanick J.I. (2000). Biochemical response to treatment of bone hyperresorption in chronically critically ill patients. Chest.

[38] Jeng L., Yamshchikov A.V., E Judd S., Blumberg H.M., Martin G.S., Ziegler T.R., Tangpricha V. (2009). Alterations in vitamin D status and anti-microbial peptide levels in patients in the intensive care unit with sepsis. J. Transl. Med..

[39] Chun R.F., Peercy B.E., Orwoll E.S., Nielson C.M., Adams J.S., Hewison M. (2014). Vitamin D and DBP: The free hormone hypothesis revisited. J. Steroid. Biochem. Mol. Biol..

[40] Passeron T., Bouillon R., Callender V., Cestari T., Diepgen T., Green A., Van Der Pols J., Bernard B., Ly F., Bernerd F. (2019). Sunscreen photoprotection and vitamin D status. Br. J. Dermatol..

[41] Amrein K., Parekh D., Westphal S., Preiser J.-C., Berghold A., Riedl R., Eller P., Schellongowski P., Thickett D., Meybohm P. (2019). Effect of high-dose vitamin D3 on 28-day mortality in adult critically ill patients with severe vitamin D deficiency: A study protocol of a multicentre, placebo-controlled double-blind phase III RCT (the VITDALIZE study). BMJ Open..

